# Network psychometrics in practice: A practical framework for designing empirical studies that utilize a psychological network approach

**DOI:** 10.3758/s13428-026-02965-7

**Published:** 2026-03-27

**Authors:** Monique Chambon, Jonas Dalege, Janneke E. Elberse, Frenk van Harreveld

**Affiliations:** 1 University of Amsterdam, Amsterdam, The Netherlands; 2 National Institute for Public Health and the Environment (RIVM), Bilthoven, The Netherlands

**Keywords:** Network psychometrics, Psychological network, Psychological systems, Study design, Framework

## Abstract

A psychological network approach enables the study of psychological phenomena as networks of interacting elements by utilizing network psychometrics. This approach is gaining increasing popularity in different domains of empirical psychological research such as clinical and social psychology. Simultaneously, a scientific debate has emerged on the added value and application of employing such an approach. The current paper contributes to this debate by providing an applied perspective on designing empirical studies that employ a psychological network approach and does so based on best practices in previous empirical psychological research. With a practical framework, we aim to support researchers who want to employ a psychological network approach to mitigate potential criticism in the design phase of their study. The framework can be summarized in three iterative steps. This paper describes each step in detail, including illustrative examples and practical considerations. First, researchers are advised to evaluate their argumentation for adopting a psychological network approach and whether this is the best approach for their research aim. This approach is suitable for aims ranging from descriptive accounts of complex survey data to testing hypotheses about the network’s structure. Second, researchers should carefully evaluate which variables the psychological network should contain. Such decisions should be informed by previous research (e.g., theoretical frameworks). Third, researchers should consider which research design and type of data are optimal to answer their research questions, as different designs provide different insights. Adopting a psychological network approach in accordance with the framework presented in this paper can further advance empirical psychological research.

A psychological network approach enables the study of psychological phenomena as networks of interacting elements (Borsboom & Cramer, 2013). The assumption underlying this approach is that psychological constructs, such as intelligence or mental disorders, are emergent properties of networks of interacting elements, as opposed to the more conventional perspective of latent constructs as common causes (Borsboom, 2017; van der Maas et al., 2017). For example, instead of depression being a latent construct that causes symptoms, depression can be modeled as a network of causally interacting symptoms, such as depressed mood, insomnia, and fatigue, that reinforce each other, with depression being an emergent property of the interacting symptoms. A psychological network approach has been applied to a diverse set of psychological constructs such as intelligence, mental disorders, and personality (Borsboom, 2017; Cramer et al., 2012; van der Maas et al., 2017).

The causal attitude network (CAN) model (Dalege et al., 2016) is a psychological network approach to attitudes. This model conceptualizes attitudes as networks of interacting attitude elements, consisting of beliefs, emotions, and behaviors. As a result, attitudes can be modeled as emergent properties of networks with interacting attitude elements (i.e., emotions, beliefs, and behaviors), instead of attitudes being latent constructs that cause attitude elements. To exemplify the application of the CAN model, Fig. 1 shows a hypothetical and simplified example of a small attitude network toward meat consumption. Such psychological networks can be modeled with methods from a field called network psychometrics (Borsboom et al., 2021; Isvoranu et al., 2022). These methods allow for estimating psychological networks with (survey) data. While several tutorial papers on the estimation and analysis of psychological networks have been published (e.g., Borsboom et al., 2021; Dalege et al., 2017a; Epskamp et al., 2018a), guidelines for designing empirical studies on psychological networks are lacking in the literature. The aim of this paper is to fill this gap.

**Fig. 1:**
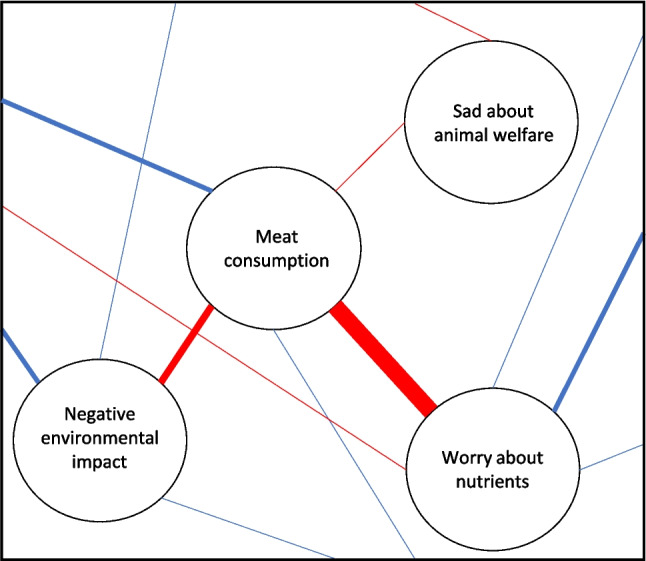
Hypothetical and simplified attitude network toward meat consumption. This hypothetical attitude network consists of a behavioral element (Meat consumption), cognitive evaluation (Meat consumption has a negative impact on the environment) and two affective evaluations (Worry about nutrient intake when reducing meat consumption/Sad about animal welfare when consuming meat). The relations can be either positive (blue/light gray) or negative (red/dark gray), and the strength of the relations is indicated by edge width. In this example, the node on worries about nutrients has a stronger negative relation with the node on meat consumption than the node on negative environmental impact has with the node on meat consumption (displayed by edge width, with thicker edges indicating stronger relations). These nodes are part of a larger network, as indicated by the edges that stretch outside the figure

In empirical networks, nodes represent the variables in the data, and edges represent (linear) statistical relations between variables. The edges indicate relations between two nodes after controlling for the effects of the other nodes in the network (i.e., conditional relations). This means edges indicate direct and unique relations between nodes that cannot be explained by other nodes in the network. Edges can represent positive (excitatory) or negative (inhibitory) relations between nodes, and their strength can differ between edges. In addition to providing insightful visual representations of survey data, the structure and properties of empirical psychological network models provide information that cannot be derived from individual relations between variables. Such properties include the overall connectivity of the network (i.e., quantifying how connected an entire network is), the centrality of nodes within the network (i.e., quantifying how connected a specific node is to the rest of the network), and which nodes tend to cluster together (i.e., providing insight into clusters of nodes that are highly interrelated).

The popularity of network psychometrics is illustrated by the vast number of scientific publications in this field over the last few years, predominantly on mental disorders (see Robinaugh et al., 2020, for a review). Consequently, guidelines on estimation methods and reporting standards have been published (Burger et al., 2023; Isvoranu & Epskamp, 2023). However, as is inherent to scientific innovation, the field of network psychometrics is also critically examined on its value beyond more conventional methods. For instance, Neal et al. (2022) published general recommendations for researchers that employ network psychometrics, such as only using such methods when it fits their research questions better than conventional methods, and refraining from causal and within-person inferences based on cross-sectional data (see also Borsboom et al., 2022). Other research has evaluated the interpretation of statistical measures derived from psychological networks (Bringmann et al., 2019; Neal & Neal, 2023). Although such critiques are inherently valuable to advance scientific research, it might be a challenge to translate such recommendations into practice. This difficulty is perpetuated by the lack of literature on guidelines for designing empirical studies that utilize a psychological network approach. This paper aims to fill this gap by presenting a practical framework that applied researchers can consult in the preparation phase of empirical psychological research in which the psychological network approach is employed. As such, this paper is aimed at applied psychological researchers who have decided they want to employ a network approach, and thus does not present an elaborate overview of advantages and disadvantages for researchers who are still considering their method. Note that the aim of this paper is not to directly refute criticism of this approach, but to provide practical guidance on how to apply this approach in a way that potential limitations are mitigated. Finally, it should be noted that as the aim of this paper is to provide applied recommendations regarding the study design, it does not discuss statistical methods underlying the network models (for the latter, readers are referred to Isvoranu et al., 2022).

The practical framework presented here is relevant for empirical research across various psychological domains. The basis for this framework lies in expertise gained in various empirical social psychological research projects that predominantly cover the domains of health, safety, and sustainability (Chambon et al., 2022a, 2022b, 2022c, 2023a, 2023b, 2023c). However, the content is substantiated with research from other domains, making the framework relevant for other psychological research domains as well, such as personality, psychopathology, and organizational psychology. Consequently, the framework is presented in broad terms that are applicable to multiple psychological research domains and illustrated with examples from predominantly attitude research.

## Practical framework for designing empirical research

**Fig. 2:**
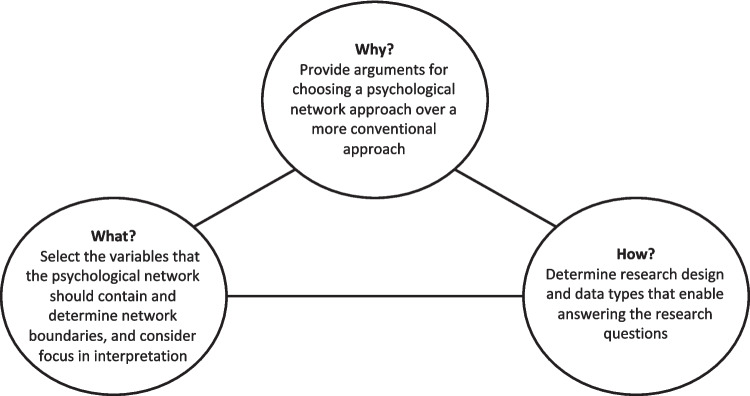
Graphical representation of three main steps to consider in future research that employs a psychological network approach

The practical framework for empirical research is presented as three steps to consider when designing a study that employs a psychological network approach (see also Fig. 2). Note that these steps are intertwined and might affect each other and should therefore not be approached as a linear process. After presenting these three steps, we present a table with a set of questions and example answers for each step to support reflection in the design phase of empirical studies (see Table 1).

**Table 1:** A non-exhaustive set of questions and example answers to support reflection in the design of empirical studies using a psychological network approach

*Questions*	*Possible answers*
Step 1: Why? Provide argumentation for adopting a psychological network approach	
*Questions*	*Example answers*
Why do I want to use a psychological network approach? What is the added value of this approach compared to simpler approaches?	E.g., modeling psychological constructs as systems of interacting elements instead of latent constructs; presenting descriptive accounts of complex survey data; graphical representation of data as networks facilitates interpretation of such descriptive accounts; insight into the relative importance of variables in the network; combining metrics on edge and network levels; data-driven, agnostic approach
Step 2: What? Select variables of interest and determine network boundaries, and consider focus in interpretation	
Which variables should I include in the network? What are meaningful network boundaries, that is, how do I determine which variables (not) to include in the network? How do I avoid redundancy in the variables in the network?	Is the topic studied extensively? Consult available theoretical and conceptual frameworks. Is it a novel topic? Consult general (psychological) models and complemented with variables based on literature from an adjacent topic
What practical considerations should I take into account?	E.g., missing data; number and type of variables and consequences for sample size and power requirements
How will I compose the nodes?	E.g., single-item nodes and/or aggregated nodes
Which point(s) of focus will I adopt when interpreting the results?	E.g., focus on one or more specific variables, or on network level metrics
Step 3: How? Determine research design and data types to answer research questions	
What type of data do I need to answer my research question? What type of data can I collect?	E.g., cross-sectional data for insights in psychological networks at the between-person level and differences between groups; longitudinal data for insights into between- and within person level networks (i.e., temporal, contemporaneous and between-person networks) and potential feedback loops; experimental data for insights into causal relations between nodes or to understand intervention effects
Which challenges unique to my research context should I take into account?	E.g., skewed data distributions; the use of diagnosis-driven scales; skip structures; subsampling based on cutoff criteria

### Step 1: Why? Provide argumentation for adopting a psychological network approach

It is important to substantiate the decision to adopt a psychological network approach. Therefore, researchers are advised to evaluate beforehand which aspects of their research plan substantiate adopting this approach. This includes questioning whether a more conventional approach could suffice. In line with this, the following paragraphs discuss different research aims for which a psychological network approach has been shown to be a suitable tool. Note that this overview is based on a subset of previous research and is therefore non-exhaustive.

#### Descriptive accounts

A psychological network approach has been shown to be useful for the aim of *presenting descriptive accounts of complex survey data*. A unique selling point is that the graphical representation of data as networks facilitates interpretation of such descriptive accounts. Note that complexity could refer to different aspects of the data, such as the number of variables, the type of variables (e.g., binary, ordinal, or continuous variables or a combination of different types), and the relations between them (e.g., conditional relations, direct or indirect relations). For example, attitude networks towards meat consumption could contain variables tapping into self-reported meat consumption, reasons to eat meat such as taste or diet being central to one’s identity, and reasons *not* to eat meat such as environmental impact, animal welfare, and health-related variables. Network psychometrics enables estimating networks with a relatively high number of psychological variables compared to conventional methods (Boutyline & Vaisey, 2017; Chambon et al., 2022a; Dalege et al., 2017a; Keskintürk, 2022; Turner-Zwinkels et al., 2021). Moreover, networks can be estimated with different types of variables, including with a combination of types of variables (e.g., ordinal and binary). Networks also provide insight into unique relations between variables, that is, relations that remain after controlling for the effects of the other variables included in the network. Consequently, psychological networks provide insight in both direct relations (i.e., an edge in the network) and indirect relations (i.e., variables that are only connected through other variables). Note that although indirect relations provide indications for mediator effects, additional research is required to establish mediation processes (Bullock et al., 2010; Rohrer et al., 2022).

It is also important to note that the data-driven nature of network psychometrics allows for an exploratory approach to describing complex survey data. This data-driven aspect is another selling point, as variables are not classified as independent or dependent up front, and researchers adopt an agnostic approach to the data. Such an agnostic approach often resembles reality when working with complex data, as it is difficult to hypothesize about specific relations between a large and/or diverse set of variables. A psychological network approach to describe multivariate data has proven useful for a diverse set of topics, ranging from relatively unexplored topics such as biobased plastic in a consumer and medical context (Chambon et al., 2023c; Zwicker et al., 2020) to novel topics that can build on comparable previous research such as the COVID-19 pandemic (Chambon et al., 2023a) and purchase intentions of sustainable electronics (Wallner et al., 2024; Zwicker et al., 2023) to topics that have been the focus of previous research such as science interest (Sachisthal et al., 2019), anti-Roma bias (Nariman et al., 2020), post-traumatic stress disorder (Armour et al., 2017), perfectionism (Di Fabio et al., 2022), and nuclear energy (Smolinski et al., 2024).

#### Variables’ relative importance

Additionally, adopting a psychological network approach provides *insight into the relative importance of variables* in the network, as this approach estimates weights of relationships between variables. In the context of meat consumption, one could examine whether, after controlling for the effects of other nodes in the network, meat consumption is more strongly related to reasons to eat meat or to reasons *not* to eat meat. Consequently, results can provide input for prioritization strategies regarding variables to focus on in future research. For instance, a theoretical framework can provide a large set of variables to include in cross-sectional research, after which a smaller set of variables can be selected for subsequent longitudinal research (e.g., see Chambon et al., 2023b, 2023c). Such relative importance of variables can be examined on different levels, which are detailed below.

First, variables’ relative importance can be indicated by their relation with the variable(s) of interest (edge level). For instance, one could examine which variables have the strongest relation with outcome variables such as behavior (e.g., self-reported meat consumption and/or signing a petition). Second, one can examine which variables are relatively important for the network as a whole (network level), which can be understood via node centrality metrics. An example of such a metric is node strength, encompassing the sum of the absolute edge weights of all edges a node has with other nodes in the network. High node strength implies that a node has many and/or strong edges with other nodes in the network, which can be interpreted as an indication of the node’s relative importance for the network. Moreover, combining the edge and network level indications of importance can provide additional argumentation for the variable’s relevance for the topic of interest, which is another unique selling point of network psychometrics. For instance, if reasons *not* to eat meat have relatively strong edges with meat consumption and also relatively high node strength, this implies reasons *not* to eat meat are more important for meat consumption than reasons to eat meat and other variables in the network. Combining results on edge and network levels can be especially valuable when working with cross-sectional data that do not provide insight into the direction of effects, because results that are consistent on different levels can substantiate each other. More specifically, with cross-sectional data, it remains unknown whether edges go *from* meat consumption *towards* other variables or vice versa, or even both directions (i.e., bidirectional edges). When aiming to improve the understanding of the network as a whole (i.e., beyond finding the strongest predictors of a single variable), results that point toward reasons not to eat meat as being relatively important for meat consumption on both edge and network levels provide stronger evidence than results that point towards relative importance on only edge or network level. To summarize, psychological networks provide valuable input for future research by indicating which more concise set of variables appears most relevant, for example, for longitudinal research with shorter surveys or for intervention studies to examine causal relations.

Some questions that other methods than network analysis (e.g., factor analysis) might be able to address can be more straightforwardly answered by network analysis. For instance, factor loadings might be used to identify influential variables instead of using strength centrality in networks. However, this can be considered an indirect approach, given that factor loadings in confirmatory factor analysis are not actually a measure of influence but a measure of how well a variable measures the underlying construct. We therefore argue that if researchers are interested in which variables have the most influence in a system of interconnected variables, they should use a measure that directly assesses this question, such as strength centrality.

Note that there are certain limitations to centrality measures that warrant discussion. First, node centrality measures do not allow for causal inferences (Dablander & Hinne, 2019). In the example of node strength (i.e., sum of a node’s absolute edge weights to other nodes in the network), cross-sectional data do not provide insight into whether high node strength implies that many and/or strong edges go *from* that node *towards* other nodes or *from* other nodes *towards* that node (or both). Second, although there are alternative centrality measures such as closeness (i.e., average of shortest path length to other nodes in the network) and betweenness (i.e., metric for a node’s position on the shortest path between two other nodes), these measures are considered unsuitable for psychological networks (Borsboom et al., 2021; Bringmann et al., 2019; Opsahl et al., 2010). Readers are referred to the work of Bringmann et al. (2019) for more in-depth information about centrality measures in psychological networks. Third, it should be noted that centrality measures depend on the network structures and thus can change as a result of variable selection and sampling variation. Therefore, node centrality measures should always be interpreted within the context of the network and its variables and cannot be generalized to other networks. Researchers are advised to conduct centrality stability analyses to evaluate whether centrality measures are stable and to assess the effects of sampling variation, and to verify whether differences between nodes’ centrality measures are significant (Epskamp et al., 2018a).

#### Other applications

It should be noted that a psychological network approach is suitable for research aims in addition to those described here. For instance, Dalege and colleagues adopted an attitude network approach in confirmatory research into the relation between the network structure and behavior (Dalege et al., 2017b; Dalege et al., 2019). Other confirmatory research focused on the relation between attitude strength and network connectivity (Dalege et al., 2019; Nariman et al., 2020). Furthermore, as demonstrated by Buttlar et al. (2023), a network approach can provide insight into nomological networks that support the process of survey validation.

A psychological network approach is probably less suitable for studies that include a very limited set of variables with the aim of identifying small sets of predictors of psychological phenomena. For instance, when investigating the relation between general attitude towards meat consumption, general health, and self-reported meat consumption to identify a single predictor of interest for an intervention aimed at lowering meat consumption, a more conventional approach might be more efficient. However, a psychological network approach can still be valuable in studying components of a broader system, even when not all variables of a system are included. For instance, insights into subsystems or partial networks can be useful when it is not possible to study the system as a whole, for example, because of a lack of variability in parts of the system (e.g., Brinkhof et al., 2023). In summary, researchers are advised to carefully evaluate why adopting a psychological network approach is preferred over more conventional approaches.

### Step 2: What? Select variables of interest and determine network boundaries, and consider focus in interpretation

When employing a psychological network approach, one can conceptualize the psychological constructs of interest as an emergent property of a network or as a system of interacting variables. This step includes determining the network’s interacting elements (i.e., nodes) and thus which variables to include in the network. As complex psychological systems are arguably limitless, it can be challenging to determine the boundaries of a network. Neal and Neal (2023) argue that it is important to construct networks with theoretically meaningful boundaries, as the selection of variables could affect measures such as node centrality. While careful consideration of variables inclusion is important for a psychological network approach, it should be noted that this challenge is not unique to network analysis but extends to regression approaches in general (i.e., omitted variable bias). There are different strategies to cope with this challenge in empirical research, which will be discussed next.

#### Approaches to variable inclusion

For *topics that have been studied extensively*, the selection of variables to include in networks can be guided by available theoretical and conceptual frameworks. For instance, in a previous study on risk perception networks (Chambon et al., 2023b), the available integrative risk perception network (Renn & Benighaus, 2013; Renn & Rohrmann, 2000) guided the selection of variables to include in the network. Another example would be to use the Diagnostic and Statistical Manual of Mental Disorders (DSM; American Psychiatric Association, 2022) to determine which symptoms to include in a network that represents a mental disorder (see for example Boschloo et al., 2015). This strategy can also be applied to parts of a network: a network on the temporal dynamics of the COVID-19 behavioral measures (Chambon et al., 2023a) was partially based on a more general framework on health behavior during pandemics (Bish & Michie, 2010). Given the new context of an unprecedented pandemic and the availability of more recent research, the variables from this health behavior framework were complemented with variables from general social cognition models such as the theory of planned behavior (Ajzen, 1991) and the health belief model (Rosenstock, 1974; Rosenstock et al., 1988) and additional variables based on more recent scientific literature.

For *novel topics*, the selection of variables can be based on general (psychological) models and complemented with variables based on literature from an adjacent topic. For example, early research into attitudes towards cultivated meat could combine variables from frameworks on meat consumption with frameworks on modified foods such as genetically modified organisms (GMOs). Novel topics are accompanied by the additional challenge of deciding which variables to include in the survey. As demonstrated in previous research in the context of attitudes (Chambon et al., 2023c; Zwicker et al., 2020), this challenge can be addressed by conducting an initial study to identify the most prevalent attitude elements. In such a study, respondents respond to open questions in which they are invited to list their thoughts, feelings, and behaviors related to an attitude object (for more information see Maio et al., 2019). Responses are then reduced to a manageable set of thoughts, feelings, and behaviors, which serve as input for developing a survey that taps into these attitude elements. This approach is similar to what is advocated by Fishbein and Ajzen (1975) in the context of the theory of reasoned action/theory of planned behavior (Ajzen, 1991). For example, in the context of meat consumption, if respondents list words such as climate change or pollution in response to the question on thoughts, one can include an item in the survey on beliefs about the environmental impact of meat consumption.

One could argue that all studies on topics for which the most prevalent psychological variables or elements of such variables have not been identified in earlier research could benefit from an initial study to identify network elements. This would extend the data-driven character of the psychological network approach to the earlier phase of variable selection as opposed to only the statistical analysis being data-driven. Note that studies designed to identify psychological variables or elements of such variables should take theoretical foundations into account. For instance, the tripartite model provided guidance for including affective, cognitive, and behavioral elements to conceptualize attitudes in the CAN model (Dalege et al., 2016). In summary, the empirical lesson here is that this step requires balancing between, on the one hand, including available theoretical and conceptual frameworks and social cognition models to substantiate variable selection and network boundaries, and on the other hand including additional variables of interest to further meet the objective of an ecological valid psychological network. Moreover, in doing so, researchers should also avoid redundancy in variables included in the network. A practical approach to this is proposed by Burger et al. (2023), who combined items when there was a strong conceptual overlap in content and the bivariate correlation between items was *r* ≥ .50. Which considerations prevail in this step depends on various aspects such as the comprehensiveness of available theoretical models and practical constraints such as those discussed next.

#### Practical considerations

Variable selection is also subject to more *practical considerations*. For instance, missing data can pose a challenge for network estimation. Network models that require correlation matrices as input can handle pairwise deletion of missing data, yet working with other models might require listwise deletion of missing data (Blanken et al., 2022). There are imputation strategies available for different network estimation methods, such as full-information maximum likelihood estimation in the R package *psychonetrics* for time series and panel data (Epskamp, 2020a). However, note that, in line with handling missing data in other statistical methods, these strategies rely on the assumption that data are missing at random (Blanken et al., 2022). Those interested in in-depth information are referred to specialized literature (Falk & Starr, 2025; Mansueto et al., 2023; Nehler & Schultze, 2024, 2025). Also, the number of variables in the network and the type of data that underlie them can affect the sample size required for accurate network estimation.^1^ That is, a higher number of nodes implies more potential edges between nodes, and detecting these effects requires a higher sample size. Blanken et al. (2022) recommend including a maximum of 30 nodes in a psychological network to keep the network interpretable and the required same size manageable, and recommend aiming for the highest sample size possible. Note that the recommendation regarding the number of nodes refers to a maximum rather than a guideline for typical psychological study designs. Given the substantial sample size requirements for psychological networks with around 30 nodes, including fewer nodes is generally preferable and more realistic for adequately powered psychological studies.

**Fig. 3:**
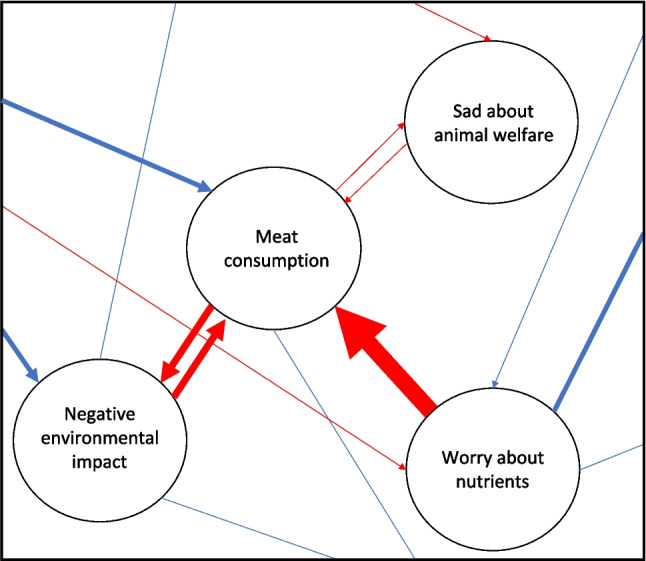
Hypothetical and simplified temporal attitude network toward meat consumption. This hypothetical network contains the same nodes as Fig. 1. Edges represent directed partial correlations and are depicted as arrows to indicate the direction of relations between nodes. Similar to cross-sectional networks, edges are either positive (blue/light gray) or negative (red/dark gray), and the strength of the relations is indicated by edge width. In this example, the relation between “Worry about nutrients” and “Meat consumption” is unidirectional from worry to consumption. The relations between “Meat consumption” and “Negative environmental impact” and “Sad about animal welfare” are bidirectional. Such bidirectional relations suggest potential feedback loops, that is, a decrease in “Meat consumption” results in an increase in “Negative environmental impact” at the next measurement, which in turn results in a decrease in “Meat consumption” on the measurement after that, and so on. Note that multiple such bidirectional relations combined can form a reinforcing structure. For instance, if three nodes have positive bidirectional relations with each other, these nodes would reinforce each other over time. Similar to Fig. 1, these nodes are part of a larger temporal network, as indicated by the edges that stretch outside the figure

Regarding the type of data, previous research showed that binary nodes might require higher sample sizes than ordinal or continuous nodes (Epskamp, 2017; van Borkulo et al., 2014). Furthermore, psychological networks based on longitudinal panel data can (currently) be modeled only with continuous or ordinal variables. Researchers should also realize that estimating temporal networks (i.e., networks with arrows indicating the direction of relations over time between nodes such as depicted in Fig. 3, which are modeled with repeated measures) is challenging when the network contains variables that show little variance over time. Therefore, temporal networks should consist of variables that are expected to change over time, so that there is enough (intrapersonal) variance in the data to detect effects. This might impose a challenge for research into psychological variables that are expected to be rather stable over time. Thus, in the case of longitudinal data collection, researchers should evaluate whether one can expect change in the variables of interest, and in which time frame, and adjust the research plan and points of data collection accordingly. Consequently, a psychological network approach might be less suitable for modeling temporal relations that include relatively stable variables such as demographics or personal values, despite their potential relevance for the topic of interest.

#### Node construction

Note that after selecting the variables of interest and converting these into survey items, one needs to consider *how to compose the nodes* that are included in the network. Psychometric networks can be estimated with single item nodes or aggregated nodes that consist of multiple items. While single-item measurements may be more prone to measurement error (de Ron et al., 2022), aggregated nodes only reduce this type of error under certain conditions (e.g., when all items are interchangeable). There are several arguments for and against both approaches (Allen et al., 2022), and researchers should determine their preferred route depending on their study (e.g., variables of interest). Combining survey items into aggregated nodes can be based on validated scales, predetermined combinations (e.g., based on theoretical foundations or when only two items covered a variable), or the results of dimension reduction analysis (e.g., principal axis factoring). When results of such dimension reduction analyses are inconclusive, decisions can be guided by theory. Therefore, a psychological network approach is data-driven in estimating the network models, but less so in the process of selecting and constructing nodes that are included in the psychological network. An alternative to using aggregated nodes is to use latent network modeling (Epskamp et al., 2017). In this approach, each node is represented by a latent variable that is estimated from several indicators. This approach is a more sophisticated way to include aggregated nodes in a network and has the advantage that the contributions of the indicators are weighted based on the factor loadings they have on the latent variable.

#### Focus point

Finally, it is recommended to *think in advance about which point(s) of focus to maintain when interpreting the results*. Interpreting the networks can be challenging due to the amount and abstractness of the information. This challenge especially applies to integrating results on an edge level with network measures such as node centrality and communities. Therefore, it can be helpful to focus on one or more specific variables (e.g., behavioral compliance, preferences) while interpreting the network. Note that it is not necessary to decide upon this at the start of a study, but considering this in advance is recommended because it could affect variable selection or even the decision to adopt a psychological network approach. For instance, if this consideration results in the insight that the aim of the study requires focusing only on one specific variable and its edges with a limited set of predictors, one might want to reconsider the argumentation for adopting this approach as discussed in step 1.

### Step 3: How? Determine research design and data types to answer research questions

In this step, we discuss the use of cross-sectional, longitudinal, or intervention data to estimate psychological networks, including the data’s fit for different research aims.

#### Cross-sectional study

Cross-sectional data allow for estimating *psychological networks* at the between-person level, which provide a descriptive account of the interplay and mutual dependence between variables on a between-person level (e.g., Beymer et al., 2022; Chambon et al., 2023c; Lee et al., 2022; Lind et al., 2024; Van der Hallen et al., 2020; Zwicker et al., 2020). Although directions of effects remain unknown, as applies to cross-sectional research in general, network measures such as network connectivity and node centrality could provide insights that transcend those derived from conventional statistical techniques. Moreover, psychological networks at the between-person level provide the possibility to combine different types of data. This has proven useful in practice, since such networks often consist of variables with different answer scales (e.g., ordinal, continuous, or binary). For example, this method allows for including different types of self-reported behavior on ordinal or continuous scales (e.g., a Likert scale or frequency counts) and observed behavior on binary scales such as donating money or not or signing a petition or not. It is important that researchers are aware that networks estimated from cross-sectional data are based on a blend of within- and between-person variance. To tease these different variances apart, one needs to collect repeated measures data with a sufficiently high sample size.

#### Group comparison

Moreover, the psychological network approach can be utilized to gain deeper insight into *differences between groups* in terms of how variables are related. Formal statistical tests allow us to examine whether significant differences in global network measures (e.g., connectivity) or specific edges can be observed between the psychological networks of two groups (van Borkulo et al., 2023). For example, one could examine whether the relation between sadness about animal welfare and meat consumption is significantly stronger in a sample of vegetarians than in a sample of omnivores. This test is currently available for comparing networks of independent and dependent samples (note that the latter has not yet been validated). There is also a method available to compare the networks of more than two groups with moderation analysis (Haslbeck, 2022; Haslbeck et al., 2021). Moreover, one can also use confirmatory network modeling to compare groups (Epskamp et al., 2022; Epskamp, 2020b). This can be done by testing whether constraining the networks of different groups to be equal results in worse model fit than estimating each group’s network without constraints. Such formal comparisons provide insight into meaningful differences and, consequently, similarities between groups. As a result, researchers can investigate which differences between groups require additional research to evaluate whether such differences should be considered by policy makers.

For examples of insights that can be derived from comparing psychological networks, readers are referred to studies that compared the perinatal depressive symptoms networks between groups of depressed and non-depressed women (Santos et al., 2017) or the networks of attitudes and preventive behaviors during the COVID-19 pandemic between two countries (Chambon et al., 2022a). Mkhitaryan et al. (2019) suggested an interesting strategy through which network comparison can provide insight into behavioral differences between groups. That is, instead of including behavior as a node in the network (e.g., registering as an organ donor), they conceptualized behavior as the emergent property of the network (i.e., behavior is the result of the interacting variables in the network). They then compared networks of people who display the behavior (e.g., registered organ donors) with people who do not (e.g., people who are not registered as organ donors).

Note that the results of network comparison provide insight into differences on a between-person level which do not necessarily apply on an individual level. Also, when comparing networks of groups, be mindful of introducing bias when subsamples are selected based on variables that are included in the network (i.e., Berkson’s bias). For example, in a clinical context, bias can be introduced when comparing psychological networks of DSM symptoms between groups with or without a DSM diagnosis when those groups are formed based on a cutoff score of those symptoms (for a more detailed discussion see Burger et al., 2023; Haslbeck et al., 2022; Ron et al., 2021).

When statistically comparing the networks of different groups, several *practical considerations* should be taken into account. First, comparing networks between groups requires these networks to contain the same nodes, as it is not possible to compare networks with different variables. This means that the survey items on which the nodes are based should be similar. Second, the sample size required to estimate reliable psychometric networks applies to each group separately. As a result, each group should in itself have a large enough sample size for network estimation. This makes it challenging to compare groups that consist of hard-to-reach respondents such as specific patient or job categories. Third, it is recommended to decide beforehand upon correcting for multiple comparisons to prevent this decision from being affected by the results. Detecting differences between networks requires sufficient power; therefore, significant differences can be considered meaningful. However, most networks contain a rather large number of edges, and testing for differences between groups for every edge in the network could inflate false positive results. A strategy to deal with this challenge is proposed by van Borkulo et al. (2023), who recommend correcting for multiple testing in confirmatory but not exploratory settings. In the case of the latter, this setting should be made explicit, and significant differences should be interpreted as hypothesis-generating. When comparing groups in an exploratory setting, without correcting for multiple testing, we recommend focusing on differences with the lowest *p* values and highest absolute edge weight difference to prevent overinterpretation of results.

#### Longitudinal study

Longitudinal panel data allow for estimating *psychological networks that include relations on both a between- and within-person level* (e.g., Brinkhof et al., 2023; Chambon et al., 2022c; Ebrahimi et al., 2021; Epskamp et al., 2018b; Fischer & Karl, 2023). These data thus allow us both to provide a descriptive account of relations on a between-person level and to model predictive effects between variables on a within-person level (i.e., temporal networks such as depicted in Fig. 3). The latter is especially suitable for insight into change in networks through insight into unidirectional and bidirectional relations between variables. This can expose potential feedback loops or reinforcing structures between variables (see Fig. 3 for an example), which can in turn inform predictions regarding intervention effects or behavior over time (i.e., how changing one variable would affect other variables). An empirical example of such a structure between variables can be found in the work of Chambon et al., (2023a), who provided insight in potential feedback loops and a reinforcing structure between variables surrounding compliance with behavioral measures during the COVID-19 pandemic. Potential feedback loops or reinforcing structures can also be expected in symptom networks of mental disorders. For example, in the context of depression, worrying is likely to increase sleep deprivation, which is likely to increase worrying (i.e., potential feedback loop). Moreover, in the case of a larger reinforcing structure with positive relations between symptoms, worrying could increase sleep deprivation, which increases depressed mood, which could in turn increase worrying. Taken together, temporal psychological networks provide deeper insight into the structure of the network.

Consequently, this deeper understanding of the network structure can provide a *systems perspective on interventions*, that is, a perspective that considers the system as a whole instead of focusing only on certain elements in a system (see also next section). More specifically, it can provide insight into which combination of variables in a psychological network may form a promising intervention target to achieve the desired change (i.e., input for a subsequent intervention study). For instance, one would expect that intervening simultaneously on multiple variables that are part of a reinforcing structure between variables induces more change in the target outcome variable(s) than intervening on one variable or a few variables that show little interplay with other variables, because the former might provide a sequence of effects. Temporal networks could also reveal structures between variables that might impede interventions. For instance, if the hypothetical network on meat consumption would have three nodes with bidirectional negative relations, this structure between variables could undermine intervention effects: when an intervention aimed at decreasing node 1 (e.g., meat consumption) results in increasing node 2 (e.g., worries), which in turn results in decreasing node 3 (e.g., sadness), this would then result in increasing node 1 (e.g., meat consumption), thereby undermining the effects of the intervention aimed at decreasing node 1 over time. Moreover, insight into the network structure provides input for simulation studies in which network manipulations can be simulated to gain insight into possible intervention effects. Note that strong predictions based on temporal psychological networks require the modeled effects to be combined with either theoretical arguments or intervention studies into causality, because longitudinal data can provide clues on but not evidence for causal effects.

An additional research aim for which temporal psychological networks can be valuable is that of *exploring polarization processes*. Two longitudinal studies on COVID-19 revealed patterns of predictive effects between psychological variables in the networks that provide insights into processes of polarization in the domain of COVID-19. In a study on compliance with behavioral measures during the pandemic, Chambon et al., (2023a) showed how potential feedback loops and a reinforcing structure between variables might explain why people strengthen their attitude in the initial direction, that is, polarize. Conversely, the absence of reinforcing structures between variables for topics that are known to cause polarization could hint toward polarization arising predominantly in an early phase of attitude formation. This was suggested as a potential explanation in research into COVID-19 vaccination intention (Chambon et al., 2022c), as intention was relatively stable, and the bidirectional predictive effects with intention and other psychological variables were not pronounced. Note that this alternative explanation only holds if the network is expected to contain the most important psychological variables, preferably substantiated with a body of literature. If this is not the case, the absence of predictive variables in the network is likely to be a more plausible explanation for the absence of bidirectional effects or reinforcing structures between variables.

#### Intervention study

The former section discussed how longitudinal research (without interventions) can provide insights for subsequent research into interventions. A less well-explored area is that of employing network psychometrics for that subsequent research, that is, *understanding intervention effects* (e.g., Blanken et al., 2019; Chambon et al., 2022b; Turner-Zwinkels & Brandt, 2022). While conventional psychological research into interventions often examines the effects of interventions on variables (i.e., node level), adopting a psychological network approach could provide new perspectives on intervention effects. For instance, one can include the intervention as a node in the network to examine the intervention’s conditional effects on other variables (Blanken et al., 2019). To illustrate, one could conduct a study with an intervention condition that includes information about the environmental impact of meat consumption and a control condition with neutral information and also include a binary node in the network that represents the condition respondents were in. The network would then display the intervention’s effects on other nodes in the network through edges between the intervention node and other nodes. In this example, a network could contain a positive edge between the intervention node and the node on negative beliefs about the environmental impact of meat consumption, indicating that the intervention condition has a higher score on these negative beliefs than the control condition.

Moreover, one could compare the psychological networks of different intervention conditions, which would provide insight into the intervention’s effects on relations between variables (i.e., edge level) or even on the network as a whole. To illustrate, one could estimate a network on attitudes towards meat consumption for the intervention condition and a network for the control condition. Next, one could examine whether there are significant differences between conditions in specific edges (e.g., is the relation between beliefs about the negative environmental impact of meat consumption and self-reported meat consumption stronger in the intervention condition than in the control condition?) but also in node centrality or network connectivity. This can be done with cross-sectional data for effects during the measurement in which the intervention was included, or with repeated measurements for insight into intervention effects over time.

Finally, as demonstrated in previous empirical work, one can explain spillover effects of interventions with networks estimated with cross-sectional data. That is, one could examine whether an intervention aimed at a specific node also results in change in connected nodes and whether changes in those connected nodes are mediated by the changes in the node the intervention was aimed at (for an empirical example, see Chambon et al., 2022b). For instance, if an intervention that was aimed at strengthening beliefs about the negative environmental impact of meat consumption also results in change in a connected node such as the belief that it is normal to eat meat, analyses could reveal whether changes in beliefs that it is normal to eat meat are the result of change in beliefs about the negative environmental impact of meat consumption (i.e., spillover effect resulting from change in a connected node). A similar approach can be adopted to examine inter-attitudinal change, that is, how change in one attitude results in change in other attitudes (Turner-Zwinkels & Brandt, 2022). These different applications of a psychological network approach to evaluate interventions can contribute to understanding and, consequently, predicting interventions’ effects.

As mentioned, a psychological network approach can provide insights that are relevant for developing interventions (e.g., longitudinal studies without intervention can provide input for intervention targets in future research) and for evaluating interventions (e.g., a network approach in studies which include interventions can improve the understanding of intervention effects). Both applications would likely benefit from more research. Regarding the former application, future research could focus on how studies that employ a psychological network approach can best inform different types of interventions (e.g., node-specific or general interventions). Regarding the latter application, future research could focus on how a psychological network approach can contribute to understanding the effects of interventions. An interesting avenue would be to examine which insights can be derived from analyzing intervention effects with this approach as compared to conventional approaches.

To conclude, a psychological network approach can be employed for a range of research aims and with different types of data and can result in diverse insights. Therefore, it is recommended that researchers evaluate beforehand how adopting a psychological network approach contributes to their goals. The steps discussed in this section could serve as a practical framework for doing so. Finally, it is important to note that results of the thought processes in the different steps can affect each other; therefore, it might be necessary to reiterate the steps.

## Discussion

Previous empirical research has demonstrated the psychological network approach to be useful for describing the complex interplay and mutual dependence between psychological variables. As this approach also comes with limitations and challenges, it has been accompanied by a scientific debate. To assist researchers who are interested in adopting this approach in their empirical research, we presented a practical framework for researchers to optimize how they employ a psychological network approach in empirical work. This way researchers can optimally utilize the benefits of this approach, namely, gaining insight into the relative importance of (groups of) psychological variables and their interplay. Moreover, this approach enables gaining insight into data patterns that improve the understanding of the interplay of psychological variables, such as bidirectional effects that represent potential feedback loops and reinforcing structures between variables.

In regard to the practical framework presented here, it should be noted that state-of-the-art knowledge on designing empirical studies that utilize a psychological network approach is continuously in flux. Consequently, methodological approaches to estimate psychological networks or compare networks between groups and accompanying practical considerations are under development as well. It should also be noted that this practical framework contains a non-exhaustive overview of recommendations in which considerations for designing empirical research that employs a psychological network approach might be missing. Future research could build on this practical framework by including additional recommendations such as strategies to evaluate a network’s completeness, that is, whether important nodes are missing.

Moreover, researchers are encouraged to reflect on whether there are conceptual or methodological aspects relevant for their specific research context that were not discussed in this practical framework. As mentioned, the basis of the framework presented in this paper lies in empirical social psychological research, and its content is substantiated with research from other domains to generalize it across psychological research domains. It could therefore be that aspects relevant for specific research domains are not discussed in this practical framework even though they should be considered when employing a psychological network approach. For example, the clinical research context might pose unique challenges that require additional recommendations (e.g., skewed data distributions, the use of diagnosis-driven scales, skip structures, or subsampling based on cutoff criteria). Accordingly, future researchers could use the current practical framework as a basis for a framework that is tailored to their specific research domain. In this way, the psychological research community could develop specific practical frameworks for designing empirical research that employs a psychological network approach for each domain of psychological research.

In conclusion, a psychological network approach appears to provide an innovative approach towards empirical psychological research. An important note is that the added value of employing such an approach depends on several aspects such as the research topic and aims, and this approach also comes with limitations and challenges. The psychological network approach thus complements available methods and is not a substitution, so adopting it should be a means to an end and not a goal in itself. This paper contributes to the scientific debate on network psychometrics by providing recommendations for researchers who want to employ a psychological network approach and does so in the format of practical framework for empirical research. In doing so, we expect to advance psychological research into topics that can benefit from a perspective that fosters systems thinking.

## Data Availability

Not applicable. No empirical data were collected during this study.

## Footnotes

1 Network accuracy refers to the network’s proneness to sampling variation (Epskamp, Borsboom, et al., 2018a).

## Code availability

Not applicable. No empirical data were collected during this study.

## Declarations

### Open practices statement

No empirical data were collected during this study.
